# Notch controls endothelial cells

**DOI:** 10.18632/oncoscience.355

**Published:** 2017-06-25

**Authors:** Juan Rodriguez-Vita, Fabian Tetzlaff, Andreas Fischer

**Affiliations:** Vascular Signaling and Cancer (A270), German Cancer Research Center (DKFZ), 69120 Heidelberg, Germany

**Keywords:** angiogenesis, angiocrine functions, endothelial cells, immune cells, Notch signaling

Notch signaling is an evolutionarily conserved pathway that controls numerous cell differentiation steps during development. Also in the adult, Notch signaling is essential to control stem cell differentiation in the bone marrow or the gut mucosa. Here, we briefly emphasize the roles of Notch during development of blood vessels (angiogenesis). Further, we highlight novel findings that indicate additional angiogenesis-independent functions of endothelial Notch signaling to orchestrate tumor progression and metastasis.

Canonical Notch signaling relies on cell-to-cell contacts that enable Notch receptor and ligand interactions, which results in proteolytic cleavage steps of the Notch receptor to release the Notch intracellular domain (NICD). The NICD translocates from the membrane to the nucleus where it binds the transcription factor RBPJ to induce the expression of cell type-specific Notch target genes [1].

**Figure 1:**
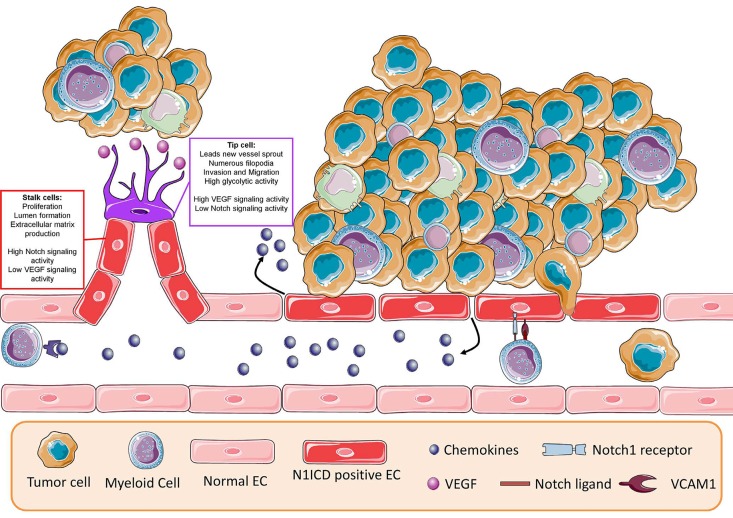
Endothelial Notch signaling regulates tumor progression VEGF secreted by tumor cells induces angiogenesis, which is controlled by Notch signaling. In existing vessels, Notch hyper-activation by tumor cells induces the expression of chemokines and adhesion molecules, which facilitate infiltration of immunosuppressive cells and the transmigration of cancer cells across the vessel wall.

Angiogenesis is induced by hypoxia and vascular growth factors, most notably VEGF, which activate quiescent endothelial cells. These cells proliferate, degrade the extracellular matrix, protrude filopodia and become migratory to induce the outgrowth of new vessel branches. Such new sprouts are guided by highly motile tip cells, whereas the trailing stalk cells form the vessel lumen (Figure 1). Notch signaling is the central coordinator of sprouting angiogenesis, e.g. by limiting the expression levels of VEGF receptors in stalk cells. Notch coordinates the selection of tip cells (low Notch signaling activity) and stalk cells (high Notch signaling activity), the number of vessel branches, vessel diameter and arterio-venous differentiation [2].

Angiogenesis is not restricted to the embryo-fetal period but also occurs in the adult, e.g. in skeletal muscle in response to exercise, expansion of adipose tissue, wound healing, or rejuvenation of bone. However, pathological angiogenesis is also a key of chronic inflammatory diseases, retinal diseases or tumor progression. It is not surprising that Notch signaling is also essential to guide angiogenesis in the postnatal period. In addition, it has become clear that Notch signaling plays an important role for the maintenance of the mature vasculature. For instance, Notch maintains the quiescent endothelial cell phenotype, the morphology and the function of the specialized sinusoidal vasculature in the liver [3].

In comparison to the normal vasculature, tumor endothelial cells often contain weaker cellular junctions and are less covered by mural cells. Hypoxia and constant secretion of proangiogenic factors from tumor cells keep many endothelial cells in an activated state resulting in a rather chaotic tumor vasculature that lacks a hierarchical structure, has irregular lumen sizes and a poorly defined arteriovenous identity [2]. Inhibition of endothelial Notch signaling using neutralizing antibodies, soluble ligands, or gamma-secretase inhibitors further aggravated this situation. The formation of excessive tip cell numbers, vessel sprouts, vessel branches and disturbances of arteriovenous identity results in a hyperdense tumor vasculature that severely lacks proper blood flow. Thereby, Notch inhibition leads to severe tumor hypoxia and tumor regression in animal models [4].

Beside these “classical” functions to coordinate physiological and tumor angiogenesis, several new angiogenesis-independent functions of endothelial Notch signaling have recently been detected. Endothelial cells do not only serve as building blocks for new vessels, but they can provide signaling molecules and secreted growth factors that instruct the behavior of parenchymal cells, e.g. during regeneration or stem cell differentiation. This has been referred to as “angiocrine functions”.[5] Such angiocrine factors also orchestrate tumor development. For instance, Cao et al., described how endothelial cells control IGF signaling in tumor cells promoting tumor progression [6]. Furthermore, Notch ligands expressed on endothelial cells can activate Notch receptors on tumor cells, which may enhance their aggressiveness and the cancer stem cell phenotype [7].

Our group has described recently how tumor cells induce Notch1 activation in endothelial cells and how this facilitates metastasis. The data revealed that endothelial Notch activation promotes expression of adhesion factors and chemokines leading to infiltration of immunosuppressive myeloid cells, migration of tumor cells across the vessel wall (Figure 1), and survival of circulating tumor cells at distant sites. This could be prevented by neutralizing antibodies against Notch1 [8].

In summary, endothelial Notch signaling is a key player during tumor progression as it coordinates angiogenesis, expression of angiocrine factors, chemotaxis, adhesion and transmigration of immunosuppressive myeloid cells and tumor cells.
